# Microbiome network traits in the rumen predict average daily gain in beef cattle under different backgrounding systems

**DOI:** 10.1186/s42523-022-00175-y

**Published:** 2022-03-28

**Authors:** Bobwealth O. Omontese, Ashok K. Sharma, Samuel Davison, Emily Jacobson, Alfredo DiConstanzo, Megan J. Webb, Andres Gomez

**Affiliations:** 1 Department of Animal Science, University of Minnesota, Saint Paul, MN 55108 USA; 2 Present Address: Department of Food and Animal Sciences, Alabama A&M University, Normal, AL 35762 USA; 3 Present Address: Community Engagement and Partnerships, Eastern West Virginia Community and Technical College, Moorefield, WV 26836 USA

**Keywords:** Backgrounding systems, Beef cattle, Rumen microbiome, Average daily gain

## Abstract

**Background:**

Backgrounding (BKG), the stage between weaning and finishing, significantly impacts feedlot performance in beef cattle; however, the contributions of the rumen microbiome to this growth stage remain unexplored. A longitudinal study was designed to assess how BKG affects rumen bacterial communities and average daily gain (ADG) in beef cattle. At weaning, 38 calves were randomly assigned to three BKG systems for 55 days (d): a high roughage diet within a dry lot (DL, n = 13); annual cover crop within a strip plot (CC, n = 13); and perennial pasture vegetation within rotational paddocks (PP, n = 12), as before weaning. After BKG, all calves were placed in a feedlot for 142 d and finished with a high energy ration. Calves were weighed periodically from weaning to finishing to determine ADG. Rumen bacterial communities were profiled by collecting fluid samples via oral probe and sequencing the V4 region of the 16S rRNA bacterial gene, at weaning, during BKG and finishing.

**Results:**

Rumen bacterial communities diverged drastically among calves once they were placed in each BKG system, including sharp decreases in alpha diversity for CC and DL calves only (*P* < 0.001). During BKG, DL calves showed a substantial increase of Proteobacteria (Succinivibrionaceae family) (P < 0.001), which also corresponded with greater ADG (*P* < 0.05). At the finishing stage, Proteobacteria bloomed for all calves, with no previous alpha or beta diversity differences being retained between groups. However, at finishing, PP calves showed a compensatory ADG, particularly greater than that in calves coming from DL BKG (*P* = 0.02). Microbiome network traits such as lower average shortest path length, and increased neighbor connectivity, degree, number and strength of bacterial interactions between rumen bacteria better predicted ADG during BKG and finishing than variation in specific taxonomic profiles.

**Conclusions:**

Bacterial co-abundance interactions, as measured by network theory approaches, better predicted growth performance in beef cattle during BKG and finishing, than the abundance of specific taxa. These findings underscore the importance of early post weaning stages as potential targets for feeding interventions that can enhance metabolic interactions between rumen bacteria, to increase productive performance in beef cattle.

**Supplementary Information:**

The online version contains supplementary material available at 10.1186/s42523-022-00175-y.

## Background

The rapidly growing world human population requires more animal protein [1, 2] and with the U.S. population projected to increase 20% by 2050 [3], an additional production of 1.7 billion kg of beef will be required to meet future demands. A major source of animal protein in the U.S. comes from beef cattle [4]; hence, producers continue to focus on improving animal genetics and feed efficiency. Improving feed efficiency would boost the feed utilization ratio, lower the amount of feed consumed, and reduce environmental impacts of beef cattle production systems [5]. As such, understanding the dynamics of nutrient cycling from feedstuff to the animal, across different animal developmental stages, is critical to improve efficiency and sustainability of beef production systems [6–8].

Generally, commercial beef production systems comprise seedstock, cow-calf, backgrounding and feedlot components. Of these four segments, the backgrounding (BKG), the period between calf weaning and placement into a feedlot [9], is vital. During this BKG period, which corresponds to the initial animal growth phase, a wide variety of feed resources are used to allow for maximal body frame and minimal fat deposition; critical characteristics that predict animal efficiency and performance at finishing stages [10, 11].

Several reports have focused on the influence of different feed sources during BKG and its duration on growth performance [12–15], highlighting diverse cost of gain and net return results depending on the system used. For instance, many producers background calves by feeding a high roughage ration in a drylot (**DL**); however, this BKG system is associated with increased labor and cost of deploying forage harvesting equipment [16, 17]. Conversely, BKG calves by grazing standing summer grown perennial pasture (**PP**) may be a more economical option due to reduced labor and greater compensatory gain when introduced to the feedlot [18]. On the other hand, BKG beef cattle on cover crops (**CC**) comprises alternative forage sources for crop-livestock production systems [11], which can protect the soil from erosion, improve nutrient cycling and increase soil productivity [19, 20]. In addition, because type of feed and environment significantly impacts feed intake, feed efficiency and animal weight gain performance; and hence, profitability of beef production [21, 22], the choice of each BKG system by producers must be carefully assessed. However, there are mixed results as far as the efficiency and applicability of each BKG system.

One way to further understand efficiency and performance of beef production systems, in the context of BKG, is to characterize the composition and ecological interactions of the vastly diverse community of microbes that inhabit the animal rumen. This rumen microbiome significantly extends the physiological capabilities of the animal; by providing access to otherwise unavailable nutrients in feed, interacting with the animal metabolic and immune landscape to impact health, and by defining the energetic efficiency and carbon footprint of the feeding process [23].

For instance, volatile fatty acids, one of the main metabolic products of microbial function in the rumen, provides the animal with up to 75% of the total metabolizable energy [6]. Propionate (mainly), as well as valerate and isobutyrate originating from bacterial fermentation of feed in the rumen, are the main substrates for do novo genesis of glucose (gluconeogenesis), an indispensable source of energy for ruminants [24]. Butyrate is the main energy source for rumen and colonic epithelial cells, which enhances their proliferation, increases growth of digestive chambers and strengthens intestinal barrier function; a key process to avoid inflammation caused by translocation of gut antigens into the gut associated immune system [25–27]. The signaling and energy harvesting roles of microbially-produced volatile fatty acids in the rumen are thus critical for optimally maintaining the immune and metabolic landscape of ruminants [28–30].

However, information on the effects of backgrounding on the rumen microbiome and its relationship with animal performance, in response to diverse BKG systems, is scarce. Therefore, the objectives of this study were to: (1) determine how the rumen microbiome of Angus and Angus x Simmental beef calves responds to diets specific to each of three different BKG systems: CC, DL, PP, from weaning to finishing; and (2) investigate associations between specific microbiome traits under the three BKG systems and growth performance through BKG and finishing. Our results indicate that diets within each BKG system drastically change rumen bacterial community composition after weaning, and that the changes observed greatly correspond to specific average daily gain (ADG). However, we show that more than the fluctuations of individual bacterial taxa, what better predicts animal growth performance, is the way bacteria interact within the rumen ecosystem. Our work sheds light on the BKG conditions that best maximize animal growth performance in beef cattle, and demonstrate that the rumen microbiome, at the community level, may be a key player in this process.

## Results

**Fig. 1:**
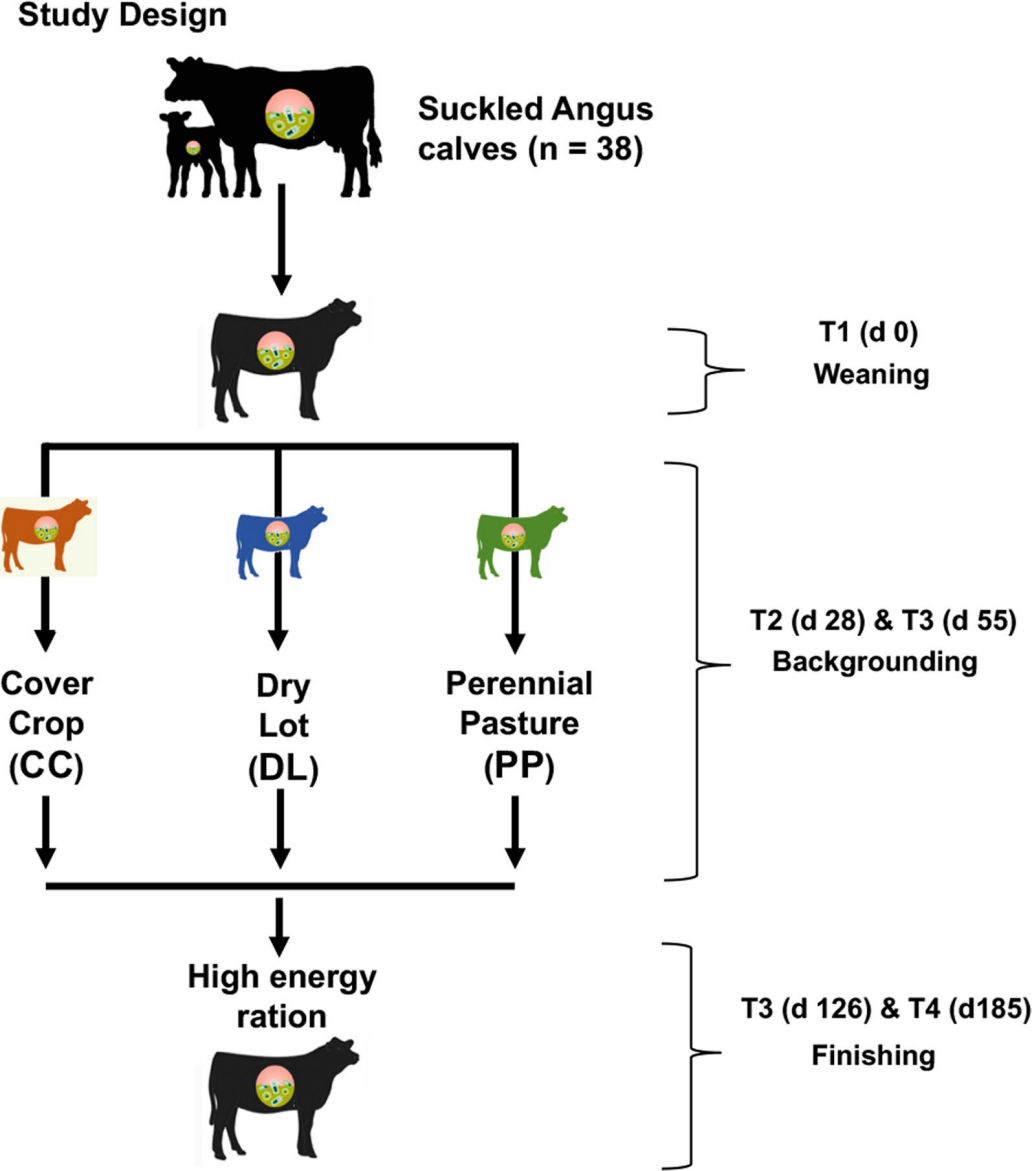
Rumen fluid sampling protocol for 38 Angus and Angus x Simmental beef calves across five points during backgrounding (BKG) and finishing. After weaning, the calves were randomly allocated to three different BKG systems: (i) dry lot (DL, n = 13); (ii) cover crop (CC, n = 13) and (iii) a third group remained grazing on perennial pasture (PP, n = 12), as before weaning. Rumen fluid samples were collected at weaning (T1), twice during BKG (T2–T3), and twice at finishing (T4–T5), when calves were kept on a high energy feedlot diet (FLD). Details on the nutritional composition of BKG and FLD diets can be seen in Table 1

Following sequencing on the Illumina MiSeq platform, a total of 5,482,279 16S rRNA short amplicon reads were obtained from 190 rumen fluid samples (amplicon libraries). After bioinformatic processing and read quality filtering, a total of 5,370,056 high quality reads remained, with a mean sequencing depth of 29,006 reads/sample (sd = 7056.38) and a total of 4149 taxonomically identified amplicon sequence variants (ASVs). From this procedure, a data frame showing the abundance distribution of each ASV (as a proxy for bacterial species or strains) across 5 time points (T) from BKG to weaning was generated (Fig. 1). Summary on feed composition and nutrient analysis during the BKG and finishing phases of the study is summarized in Table 1.

**Table 1:** Nutrient composition of backgrounding diet (% of DM) fed to cattle

Item	Backgrounding^1^			Finishing^2^
Nutrient composition^3^	DL	PP	CC	ALL^4^
Moisture	41.2	80.3	91.7	37.9
Dry matter	58.8	19.7	8.3	62.1
NEm, Mcal/kg	1.6	1.5	1.6	1.9
Neg, Mcal/kg	1.06	0.9	0.97	1.4
Starch	31.4	–	–	39.6
NDF	33.4	47.7	28.1	25.3
CP	12.6	21.3	19.5	13.4
Fat	4.3	3.6	2.6	4.9
Ca	0.6	0.6	1.3	0.6
K	1.8	2.6	4.1	1.0
Mg	0.2	0.2	0.2	0.2
S	0.1	0.2	0.5	0.2

^1^During backgrounding, animals were allocated to either one of three treatments; DL (calves were fed a haylage ration in dry lot), PP (calves grazing perennial pastures) or CC (calves grazing summer grown cover crop) for 55 d

^2^During finishing, all animals received four concentrate-adaptation diets over a period of 28 days

^3^Calves received free-choice minerals (Wind & Rain, Purina Animal Nutrition LLC, MN) during the backgrounding phase and nutrient analysis conducted on weekly feed samples

^4^ALL = calves backgrounded in CC, DL and PP were fed a similar high energy ration

### Broad taxonomic composition of rumen bacteria in beef calves during backgrounding (BKG) and finishing

**Fig. 2:**
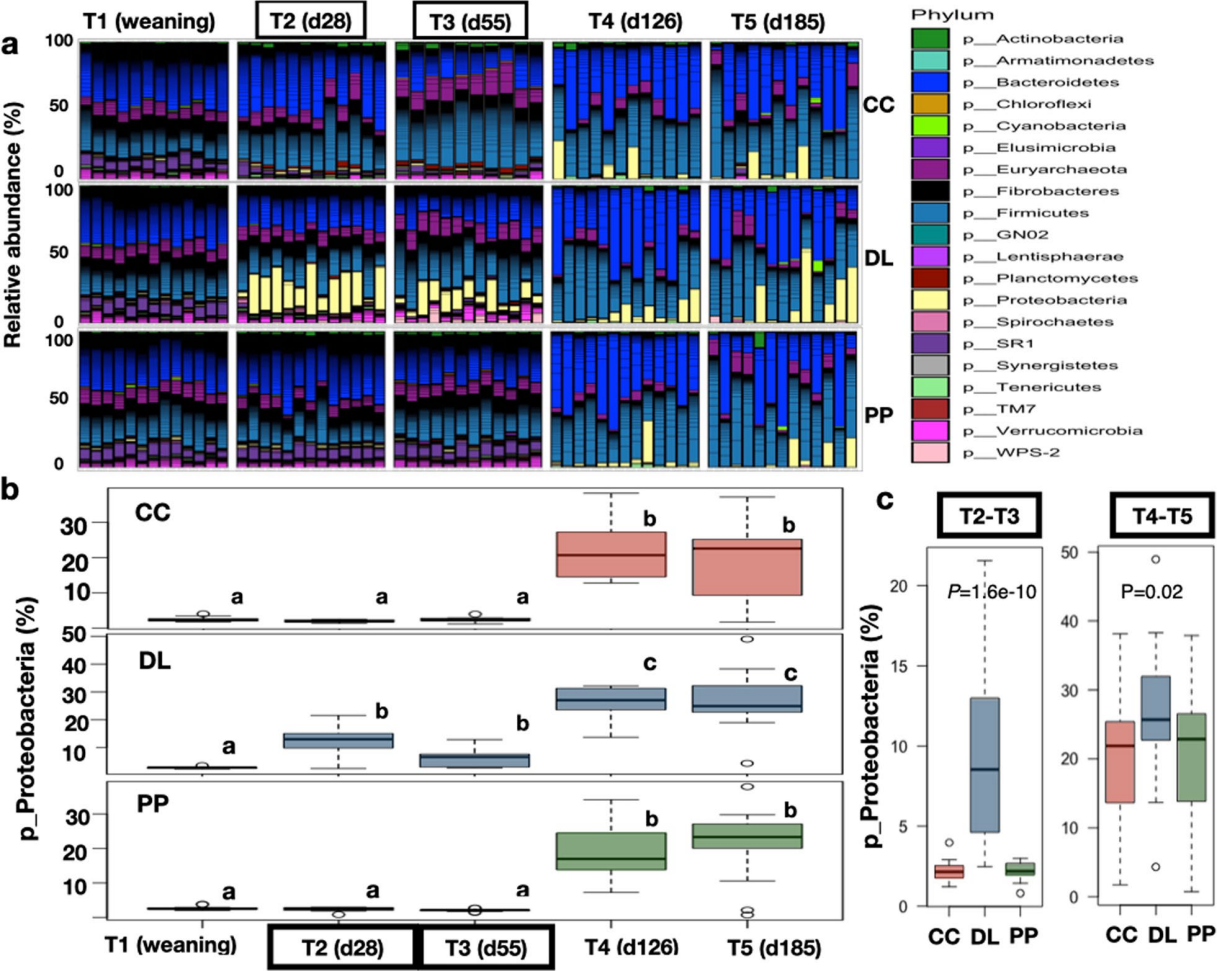
Taxonomic composition at phylum level in the rumen microbiome of calves at weaning (T1), backgrounding (T2–T3), and finishing (T4–T5). **a** Barplot showing the relative abundance of phyla at weaning, backgrounding and finishing. **b** Boxplots showing relative abundances of Proteobacteria in calves within every BKG system, from weaning to finishing. **c** Boxplots showing relative abundances of Proteobacteria in calves within specific time points, BKG:T2–T3 and finishing: T4–T5. Dry lot (DL); cover crop (CC) and perennial pasture (PP)

Rumen bacterial communities in the 38 calves during backgrounding (BKG) and finishing were mainly assigned to seven broad taxonomic groups: Bacteroidetes (37%) and Firmicutes (35%) were the two most abundant phyla, followed by Proteobacteria (12%), Verrucomicrobia (3%), Actinobacteria (2%), Tenericutes (1.5%), and SR1 (1.2%). Euryarchaeota (4%) and Crenarchaeota (< 1%) were the two archaeal phyla detected. However, significant distinctions in the abundance distribution of specific phyla between calves on each BKG system and at finishing were evident (Fig. 2a). For instance, one of the main distinctive taxonomic traits observed during BKG, was the predominance of the phylum Proteobacteria in DL calves (9.24% ± 5.15), compared with calves in CC (2.19% ± 0.61) and PP (2.23% ± 0.52) (Fig. 2b, c, Kruskal–Wallis, *P* = 1.6e−10). It was also noted that Proteobacteria, was predominant at the finishing stage (T4 and T5) compared to BKG in all three groups; however, the abundance of Proteobacteria remained the highest in DL calves during this stage (Fig. 2c, Kruskal–Wallis, P = 0.02).

### Rumen bacterial diversity was significantly impacted by backgrounding and finishing

**Fig. 3:**
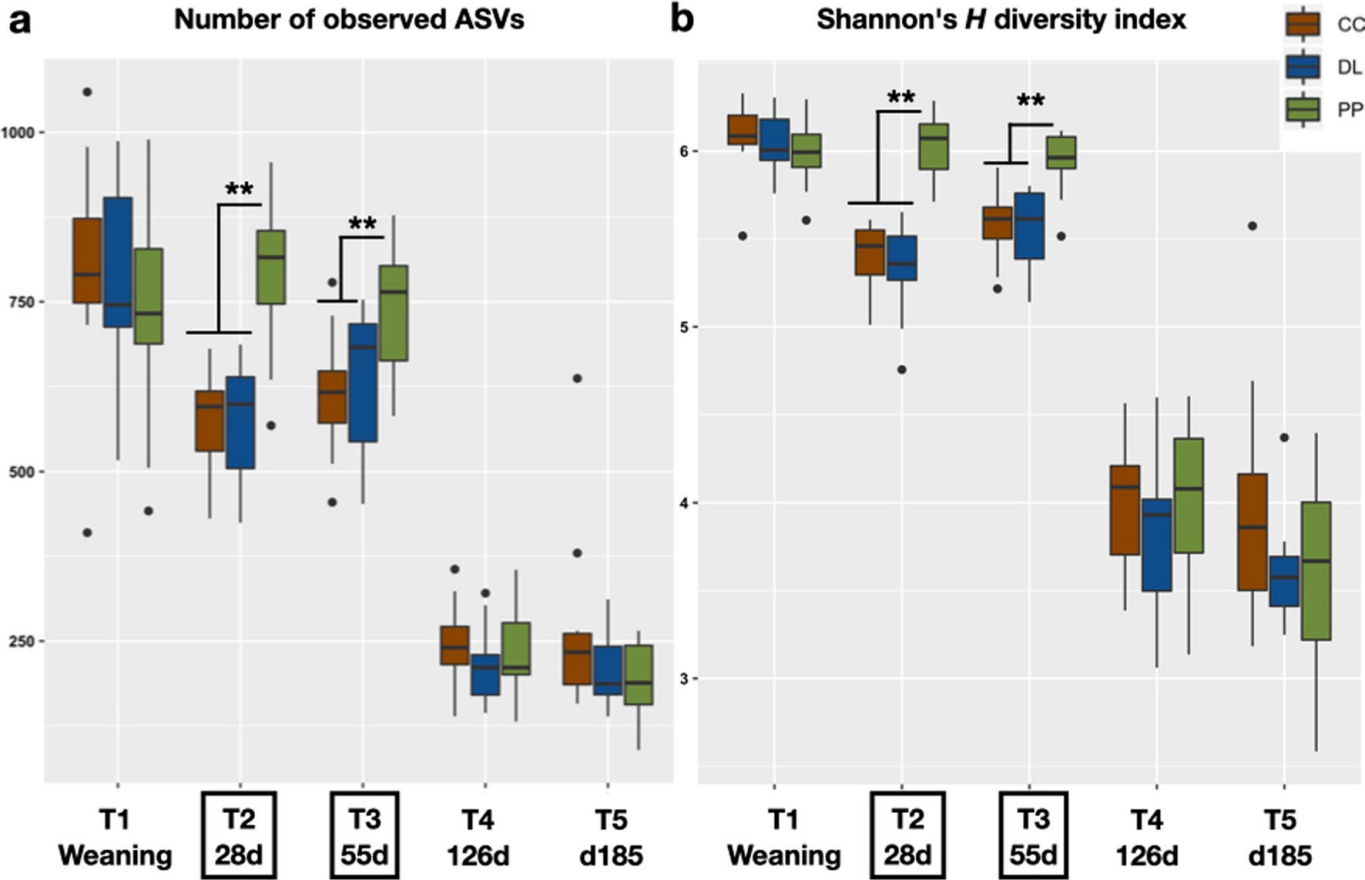
Rumen bacterial alpha diversity at weaning (T1), backgrounding (T2–T3), and finishing (T4–T5). **a** The number of different ASVs detected decreased significantly for calves moved to DL and CC BKG, but remained stable for calves on PP during this period. This number decreased again for all BKG groups at the finishing stage. **b** The patterns observed with the number of different ASVs as shown in (**a**) were replicated when measuring the Shannon index of diversity, which not only takes into account presence or absence of different ASVs, but also their abundance distribution. Dry lot (DL); cover crop (CC) and perennial pasture (PP). Asterisks show significant differences based on Kruskal–Wallis tests adjusted for multiple comparisons (**P < 0.001, ***P < 0.0001)

Alpha diversity analyses revealed significant changes in bacterial diversity during backgrounding and finishing. After weaning, for calves moved to CC and DL BKG (T2 and T3), the number and proportion of different bacterial strains or species (observed ASVs and Shannon diversity index) decreased significantly (Kruskal–Wallis test, *P* < 0.001), while remaining relatively stable for PP claves. This pattern was expected as PP calves remained on the same diet after weaning and during BKG. However, once DL, CC and PP calves were moved to the finishing phase (T4 and T5), alpha diversity decreased sharply (Kruskal–Wallis test, *P* < 0.001) for all groups, with no differences being retained between calves coming from each BKG system, at early or late finishing (Fig. 3a, b).

### Different BKG systems and the finishing stage are associated with unique rumen microbiome profiles

**Fig. 4:**
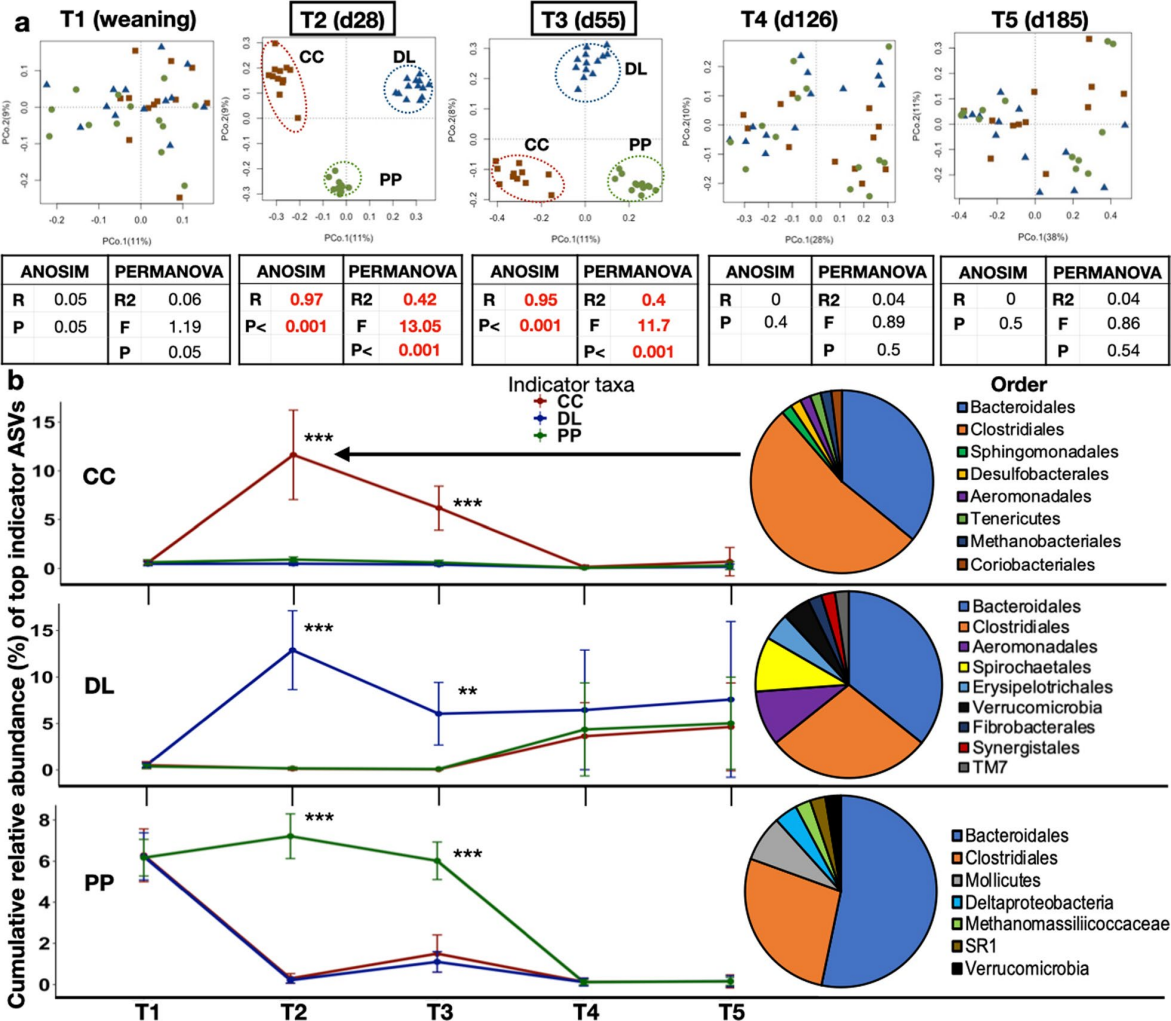
Rumen bacterial composition and abundance of indicator taxa at weaning (T1), backgrounding (T2–T3), and finishing (T4–T5). **a** Principal coordinate analysis (PCoA, Bray–Curtis distances) showing bacterial compositional differences at weaning, BKG and finishing. ANOSIM and PERMANOVA test results, along with their statistics are displayed below each PCoA plot. **b** Cumulative abundance of indicator ASVs, distinguishing each BKG system, at weaning, BKG and finishing. The line plots show how the cumulative abundance of these indicator ASVs peaks during BKG (T2). The pies show the taxonomic affiliation, at the order level, of the indicator ASVs. Asterisks show significant differences in the abundance of indicator taxa between groups based on Kruskal–Wallis tests adjusted for multiple comparisons (**P < 0.001, ***P < 0.0001). Dry lot (DL); cover crop (CC) and perennial pasture (PP)

Next, similarities and differences in the presence of specific rumen bacterial taxonomic groups (ASVs) and their relative abundances (%), during BKG and finishing were considered. This beta diversity analysis showed that, after weaning [day 28 (T2) and day 55 (T3)], each BKG system was characterized by very unique rumen microbiome profiles (Fig. 4a, ANOSIM’s R > 0.9; PERMANOVA’s R2 > 0.4 and F > 11.7, *P* < 0.001). However, once at finishing [day 126 (T4), day 185 (T5)], these differences were not maintained, likely reflecting that all calves were under the same high energy feedlot diet.

The data also show that although the major dietary changes characterizing BKG and finishing had the strongest effect on the rumen microbiome of all calves (PERMANOVA’s R2 > 0.43 and F > 23, *P* < 0.001), intrinsic drivers such as developmental stage or age were also significant factors, particularly during finishing (Additional file 1: Fig. S1). For instance, although it seemed that the rumen microbiome of calves remained unchanged under the same diet during BKG, closer inspection of the data shows significant compositional shifts from day 28 to 55 (ANOSIM’s R > 0.65, P < 0.001), with less pronounced changes from day 126 to 185 at finishing, particularly for PP claves (ANOSIM’s R = 0.16–0.21, P = 0.006–0.02) (Additional file 1: Fig. S1a). In addition, it was observed that the rumen microbiome of calves exhibited increased interindividual variability along with age, with higher heterogeneity in microbiome profiles between individuals at the finishing stage (day 126–185) (Kruskal-Wallist test P < 0.05) (Additional file 1: Fig. S1b).

Subsequently, we sought to mine for taxonomic features characterizing each BKG system. A species indicator analysis was used to mine for specific ASVs that were unique to, and more abundant in each BKG group once calves were weaned (T2 -day 56, Indicator value > 0.7, FDR-adjusted, Kruskal–Wallis test; P < 0.001, Additional file 2: Table S1). These analyses revealed that 54, 42 and 78 indicator ASVs faithfully characterized calves on CC, DL and PP BKG respectively. Although the majority of indicator ASVs across all three BKG systems were affiliated to the Clostridiales and Bacteroidetes orders (mainly Prevotellaceae and Lachnospiraceae families), the proportions of these taxa differed in each system, with other taxonomic groups also showing unique patterns (Fig. 4b, Additional file 2: Table S1).

For instance, most indicator ASVs characterizing calves under CC BKG were largely affiliated to the Clostridiales (unclassified, Lachnospiraceae), with contributions from ASVs classified as *Methanosphaera* (Archaea-Methanobacteriales), Coriobacteriaceae (Actinobacteria), *Sphingomonas*, *Desulfobulbus*, Succinivibrionaceae (Proteobacteria), and Mollicutes (order RF39). The vast majority of indicator ASVs characterizing calves under PP BKG were largely assigned to the Bacteroidales order (unclassified, BS11 and Prevotellaceae), also with contributions by ASVs classified as Methanomassiliicoccaceae (the *vadinCA11* genus), Deltaproteobacteria (orders GMD14H09, Myxococcales, ands PB19), Mollicutes (unclassified Anaeroplasmataceae, *Anaeroplasma* and 4 ASVs from the RF39 order) and Verrucomicrobia (RFP12 order).

Calves under DL BKG were characterized by lower abundance of Clostridiales, but particularly, by higher abundance of ASVs from the Succinivibrionaceae family (Anaeromonadales order, Phylum Proteobacteria); specifically, *Ruminobacter, and Succinimonas* were significantly enriched in DL calves compared to any other group during BKG (Additional file 1: Fig. S2). Other indicator taxa distinguishing DL BKG were from the Spirochaetales order (specifically, unclassified *Treponema*), unclassified Erysipelotrichaceae (RFN20), Verrucomicrobia (RFP12) and ASVs from the order Synergistales and TM7 (F16 family). Figure 4b shows that the cumulative relative abundance of these indicator taxa (classified at the order level) peaks at day 28 (T2), the first BKG time point analyzed, diminishing sharply through day 55 of BKG and finishing, except for indicator ASVs for calves under DL BKG, probably due to the prevalence of Aeromondales also at finishing. The abundance of indicator taxa in PP calves remained constant through T3 after weaning, showing the lack of dietary change previous to finishing (T3-T4). Indicator ASVs at day 28 of BKG can be seen in Additional file 2: Table S1.

### Calves display unique co-abundance network traits under each BKG system

**Fig. 5:**
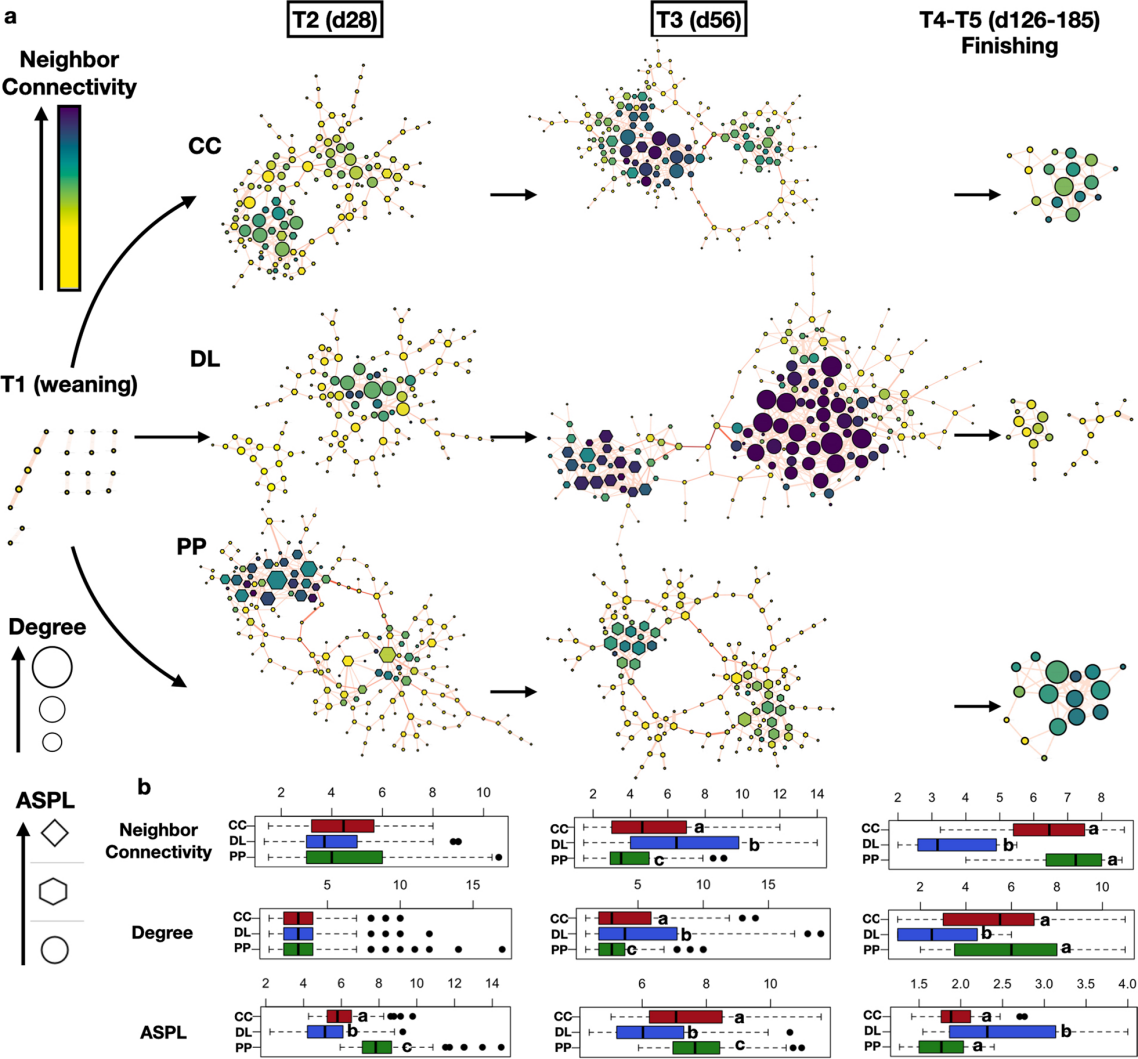
Co-abundance networks of rumen bacterial taxa at weaning (T1), backgrounding (T2–T3), and finishing (T4–T5). **a** Network topology and attributes in the rumen microbiome of calves at weaning, BKG and finishing can be visualized. Nodes represent a given ASV and edges show the association (correlation) between two given bacterial taxa (nodes). Color key represents neighbor connectivity, which measures the average connectivity of all surrounding nodes in the network. Size of node represents the average degree of connectivity or number associations a given node has in the network. Shape represents the average shortest path length, a measure of how fast information can travel through a network. Differences in the variation of all these network attributes between each group at BKG and finishing can be observed in the box plots depicted in panel (**b**), where letters represent significant differences based on Wilcoxon Rank Sum tests (P < 0.05). Dry lot (DL); cover crop (CC) and perennial pasture (PP)

The manner in which individual bacterial species co-abound or interact within a microbiome could be used as a proxy for the functional relevance and potential metabolic contribution of microbes in a given ecosystem [31, 32]. With that premise, we characterized co-abundance dynamics of specific rumen bacterial taxa (ASVs), when calves were moved from weaning to each BKG system and to the finishing stage, and inferred their collective functional potential using network-theory approaches [33]. For instance, at weaning, the rumen microbiome of all 38 calves showed extremely low connectivity (or number of interactions), with only 11 sparse interactions detected (compositionally corrected Spearman’s correlation coefficient > 0.7, q < 0.05) (Fig. 5a). This observation is likely a reflection of a very immature rumen microbiome, when reliance on lactation still constitutes the main feeding source. However, when calves were moved to each BKG system, interactions between rumen bacteria became more complex (from 193 to a maximum of 545 interactions at day 56 of BKG for DL calves, Fig. 5a and Additional file 1: Fig. S3).

To investigate if these complex rumen bacterial interactions are unique to each BKG system, we measured and compared several network attributes across BKG and finishing. For example, Fig. 5a, b show that average neighbor connectivity and degree, two centrality measures denoting the number of local and wide (direct/indirect) interactions between rumen bacteria [34], were significantly higher for calves on DL and lowest in calves on PP, particularly at day 56 of BKG (T3) (Kruskal–Wallis multiple comparisons, P < 0.001). Conversely, the average shortest path length, a proxy for functional distance between bacterial taxa (or how fast information moves through a network [35]), was always the lowest in DL calves and the highest under PP BKG at day 28 (T2) and 56 (T3).

At finishing (days 126 and 185), although all calves were under the same high energy diet, and overall alpha and beta diversity patterns distinguishing groups during BKG were not conserved, network dynamics were also significantly different, not only compared to the BKG stage, but also among calves coming from each BKG system (Fig. 5). For instance, although the number of rumen bacterial interactions decreased dramatically under the finishing diet in all calves coming from CC, DL and PP BKG (35 in DL calves to around 50 for CC and PP groups, Additional file 1: Fig. S3), the patterns observed at BKG were reversed; specifically, the rumen microbiome of DL calves showed the lowest average neighbor connectivity and degree centrality, and the highest average shortest path length compared to PP and CC calves (Fig. 5a, b). Overall network topology traits, considering these and other network attributes simultaneously (closeness centrality, clustering coefficient, eccentricity and radiality), showed that networks from DL calves were the most unique and different during BKG (T2) and finishing (Additional file 1: Fig. S4a).

Next, we mined for the most significant interaction patterns between taxa, measured as the average strength of correlation (Spearman *Rho* coefficients) and average number of shortest paths that go through a given interaction (Edge betweenness). These analyses revealed that although average correlation strength between bacterial taxa (Spearman’s *rho*) was largely similar in CC, DL and PP claves during BKG (mean: 0.75–0.77,), rumen bacterial communities in PP claves showed the greatest correlation strength at finishing (Additional file 1: Fig. S4b). In line with the average shortest path length patterns mentioned above, edge betweenness was the lowest in DL calves during BKG (d28 and d56); but at finishing, it was PP calves that showed the lowest number of shortest paths that go through a given interaction.

### Average daily gain (ADG) differed in each BKG system and at finishing

During BKG, ADG was the greatest (*P* < 0.05) in DL calves, particularly compared with calves under PP (1.4 vs 0.9 kg/d). Indeed, PP claves showed the lowest ADG during this stage. However, during the finishing stage, PP calves showed a compensatory ADG, with significantly higher values compared with DL calves (1.9 vs 1.6 kg/d*, P* = 0.02,). Overall, during finishing, ADG was greater in PP calves (1.6 kg/d), followed by CC claves (1.5 kg/d), with calves under DL showing the lowest values (1.4 kg/d), when averaging all data across all study stages (Table 2).

**Table 2:** Average daily gain (ADG) observed in calves under each backgrounding system at backgrounding, finishing and throughout the study

ADG	Treatment^1^			
	DL	PP	CC	*P*-value^2^
Backgrounding	**1.4 ± 0.1**^**a**^	0.9 ± 0.1^b^	1.2 ± 0.1^ab^	< 0.001
Finishing	1.6 ± 0.1^b^	**1.9 ± 0.1**^**a**^	1.7 ± 0.1^ab^	0.017
Overall	1.4 ± 0.0^a^	**1.6 ± 0.0**^**b**^	1.5 ± 0.0^ab^	0.021

Bold font highlights the highest ADG values obtained

^a,b^Least squares means within a row with different superscripts differ (*P* ≤ 0.05)

^1^Calves were stratified by birth date, birth weight, gender and dam age to 1 of 3 treatments: (1) dry lot (DL, calves were fed a haylage ration in a dry lot); perennial pasture (PP, = calves grazing perennial pasture); and cover crop (CC, calves grazing summer grown cover crop) for 55 d

^2^Probability of a difference among least squares means

^3^Measurements indicated are averages of data collected over the entire study period

## Discussion

Although others have demonstrated the impact of backgrounding systems on performance characteristics of beef cattle [12–14], this was the first study, to the best of our knowledge, to evaluate the effects of backgrounding on rumen microbiome structure from weaning to finishing. This report demonstrates that rumen bacterial profiles in BKG predict ADG at both BKG and finishing. We place importance on the influence of rumen bacterial communities on growth performance, not only in terms of the possible contributions of specific rumen bacterial taxa, but particularly, on how interactions between rumen bacteria impact performance depending on the BKG system selected.

### The rumen microbiome is unique under specific BKG systems and finishing in beef cattle

During growing stages, the rumen microbiome plays a critical role in feed efficiency by harnessing energy from feed for muscle development, through fermentation and protein synthesis [8, 36]. In many beef production systems, BKG is characterized by a change from calves suckling dams in addition to grazing pastures to a high-fiber, low-energy diet before final transition to a feedlot where calves receive a low-fiber, high-energy diet [37]. However, depending on the specific BKG diet, different rumen microbial profiles and fermentation activities would be expected, which may have a direct impact on growth performance and efficiency [38, 39].

In this study, it was clear that each BKG system resulted in unique rumen bacteria profiles after weaning. In fact, specific taxonomic groups dominated when calves faced drastic dietary changes in BKG and finishing, causing a sharp drop in overall alpha diversity (Figs. 2, 4). This drop in diversity is attributed to the specialized diets provided during BKG (DL and CC specifically) and finishing, characterized by the availability of specific feed substrates, readily metabolizable by selected taxa [40, 41]. The availability of specific dietary fractions, in terms of readily metabolizable energy (e.g. high starch) or fiber (NDF-ADF) may have selected for blooms and/or suppression of specific bacterial taxa, which under each new condition, along BKG and finishing, would compete for preferred and/or available energy sources.

For example, one of the most remarkable observations was the bloom in taxa of the Succinivibrionaceae family observed in calves under DL BKG, specifically the increase in the abundance of *Ruminobacter* and *Succinimonas* (Additional file 1: Fig. S2, Figs. 2, 4). Succinivibrionaceae are reported to be core taxa in beef cattle and one of the most functionally active bacteria during growing and finishing stages, as revealed by metatranscriptomics approaches [8, 42]. Members of this family, including *Selenomonas,* and *Succinivibrio,* have been observed to increase in the rumen of steers under diverse conditions; for example, when suppressing methanogens using encapsulated nitrates, with increasing the content of readily available energy in the diet ( e.g. such as those dominated by starch), and in positive association with ADG and feed efficiency[43–46].

Succinivibrionaceae are known amylolytic bacteria, which have an increased capability to produce succinic acid and formate from glucose fermentation; they can also utilize CO2 as main substrate, particularly favoring the production of propionate, which is the main gluconeogenic substrate for energy synthesis in the liver [24], besides being frequently associated with higher feed efficiency ruminants [46–48]. Such metabolic outcomes can also be explained by Succinivibrionaceae’s ability to compete for electron sinks used as substrates for the generation of volatile fatty acids other than propionate, by suppressing cellulolytic bacteria [49, 50], and antagonizing with methanogens in the rumen [51]. These observations are consistent with the taxonomic results shown here as far as suppression of Clostridiales in the DL diet and its high starch content, which is rapidly fermented in the rumen by microbes of the Succinivibrionaceae family [47]. Thus, the availability of readily degradable starch in DL BKG diets could have favored greater ADG observed in these calves during BKG, mediated by the blooms in Succinivibrionaceae and their metabolic products.

However, increasing the availability of easily fermented starch in the rumen can have detrimental consequences for the animal, by favoring disorders such as sub-acute rumen acidosis (S ARA)[52]. SARA can damage the rumen epithelium, decrease blood pH, cause dehydration, laminitis, liver abscesses and low feed intake, among other conditions [53]. Because the metabolic products of taxa such as Succinivibrionaceae may inhibit growth of cellulolytic bacteria, including butyrogenic taxa [49], the beneficial effects of such metabolites for rumen epithelial function may also be lost. This observation could help explain some of the bacterial co-abundance patterns reported here (Network analyses), and their correspondence with the differential ADG seen under each BKG system and the finishing stage.

### Bacterial co-abundance network traits predict ADG at BKG and finishing in beef cattle

An increase in highly and readily degradable starch in the rumen of DL calves could also explain the unique co-abundance network patterns observed in this group during BKG, in contrast with CC and PP calves (Fig. 5). Analyses of network centrality measures can denote the ability of microbial communities to functionally respond to external and internal stimuli [31, 32]; in this case, substrate availability. As such, a high starch degradation rate in DL calves may have altered metabolic interactions between rumen taxa, which upon release of specific metabolic products (e.g. Succinivibrionaceae-derived formate or succinic acid) could have increased the number of metabolic associations surrounding a given taxon (neighbor connectivity and total number of interactions), boosted the number of metabolic interactions (degree) and decreased the number of interactions or steps that would take to metabolically connect all possible pairs of rumen taxa (edge betweenness and path length) (Fig. 5 and Additional file 1: Fig. S5). According to concepts in network theory, the microbiome of DL calves could have been more efficient in transporting information throughout microbial metabolic networks in the rumen during BKG (e.g. towards the generation of propionate), likely positively influencing increased ADG at this stage as mentioned above.

Thus, it can be hypothesized that, microbial interactions, and hence microbiome network traits in the rumen, can predict animal physiological performance more accurately than the presence, bloom or suppression of specific taxa. For example, calves under PP BKG not only had the lowest BKG ADG, but also opposite network traits compared to those seen in DL calves during BKG. However, once at the finishing stage, when all alpha and beta diversity traits were largely the same for all groups and despite the fact that all calves were under the same high energy diet, PP calves not only showed greater ADG (significantly higher than DL calves), but also greater interaction strength, lowest number of average path lengths and edge betweenness (Fig. 5 and Additional file 1: Fig. S5). These traits were also observed despite the fact that DL calves retained the highest abundance of Succinivibrionaceae at finishing, which likely influenced the higher ADG in these calves during BKG (Additional file 1: Fig. S2).

The greater ADG of PP calves during finishing can be attributed to compensatory gain resulting from initial slower growth of PP calves during backgrounding. Research suggests that compensatory growth is influenced by nutrient restriction, including type of nutrient being restricted, the length of nutrient restriction and the type of diet fed following restriction [54]. Our data reveal that microbiome composition, and particularly, the specific characteristics of microbe-microbe interactions or network traits are also associated with compensatory weight gain. Thus, during BKG, the rumen of PP calves could have exhibited smaller net energy available to rumen microbes due to the high fiber content in pastures and restriction of starch (Table 1), leading to less dynamic rumen microbiome networks, but favoring the activity of taxa typically associated with the generation of health-producing metabolites (e.g. butyrate). This observation supports the dominance of Clostridiales and Bacteroidales in PP calves during BKG; these clades include taxa able to display greater metabolic versatility in ruminants when tackling high fiber diets (e.g. *Butyrivibrio, Ruminococcus, Prevotella*), but that exhibit different metabolic outputs compared with taxa better suited to metabolize high energy diets (e.g. Succinivibrionaceae) [23, 36, 55, 56].

Therefore, an adaptation to high fiber diets in PP calves during BKG, including a slower metabolic turnover in the rumen, could have *programmed* their rumen environment to maximize energy harvest and growth at finishing. The mechanisms behind these delayed responses cannot be elucidated with these data, but high forage during BKG, at the expense of high energy for growth, could have enhanced health by positively modulating growth of epithelial cells in the host rumen mucosa [57]. A high fiber diet during BKG could have also triggered a carryover effect on the rumen microbiome, preparing PP calves to better tackle high energy diets potentially prone to metabolic distress at finishing [40, 42, 58]. Based on the observation that the network attributes of PP and CC calves and ADG patterns were more similar during BKG (Table 2 and Additional file 1: Fig. S5), compared to those seen in DL calves, it can be speculated that a forage based diet during this growing stage, either based on perennial pastures or cover crops, achieves analogous microbiome modulation and physiological outcomes in beef cattle at finishing, independent from taxonomic assortment.

### Study limitations

The main limitation of our study is the absence of data supporting actual metabolic responses to each of the three BKG systems. As such, the results obtained should be validated using actual functional data from the rumen microbial communities evaluated, included but not limited to metabolomic and metagenomics approaches at BKG and finishing. However, these compositionally-based results reflect previous data on the associations between active rumen taxa, as measured by RNAseq, and physiological performance in beef cattle [59, 60]. Shorter rumen fluid collection interval following allocation into different backgrounding systems and during the finishing phase could also provide a better understanding of how rumen microbial communities, and associated metabolic products, begin to change under the influence of specific BKG diets. Finally, we also acknowledge that although the per-group sample sizes considered herein are customary of microbiome studies of similar scope [8, 40, 61], the performance traits reported (ADG), in the context of microbiome composition and rumen metabolites during BKG, should be evaluated and validated based on large within group populations.

## Conclusions

Our data show that specific rumen microbiome traits, and particularly, patterns of interactions between rumen taxa, can predict growth performance in beef cattle at BKG and finishing stages. Specifically, increasing dietary energy during BKG may only temporarily affect energetic turnover in rumen microbial populations and growth performance. In contrast, keeping calves under more cost-effective BKG systems such as pastures or cover crops, which enhances fiber degradation during BKG, may prove to be more effective on growth performance in the long run. The implication of these results is that producers could employ targeted feeding strategies at early life to modulate the rumen microbiome of their herd and program feed efficiency in subsequent production stages. However, given the dynamic nature of the rumen microbiome in early developmental stages, it is likely that the window for microbiome modulation needs to be carefully selected, even when calves are under the same diet (Additional file 1: Fig. S1). Finally, these data highlight the need to focus attention beyond taxonomic markers of animal performance, to focus on metabolic interactions between taxa and microbial network traits as more accurate markers of physiological performance in microbiome studies focusing on animal production systems.

## Materials and methods

### Animals and experimental design

All animal care and experimental protocols were approved by the University of Minnesota Animal Care and Use Committee (approval number 1807-36177A). This study was conducted at the North Central Outreach Research Station (NCROC), University of Minnesota, Grand Rapids, MN. A total of 38 Angus and Angus x Simmental beef calves comprising steers (n = 18) and heifers (n = 20), bred in the same location were enrolled in the study. Calves were born within a 23-d period with average birth weight 35.5 kg from dams bred at NCROC. Cow-calf pairs grazed a mix of introduced pasture grasses typical of a Northern mixed prairie comprising ryegrass (*Lolium perenne*), quackgrass [*Elymus repens* (L.) Gould], orchardgrass [*Dactylis glomerata* (L.)], smooth bromegrass [*Bromus inermis* (L.)], red clover [*Trifolium pretense* (L.)], and alfalfa [*Medicago sativa* (L.)] throughout the pre-weaning period. All calves were fence-line weaned for 6 d prior to enrollment in the study and vaccinated using a bacterin-toxoid against clostridial diseases (Ultra Choice 8, Zoetis, Parsippany, NJ) and a modified live vaccine for prevention of respiratory viruses and Mannheimia Haemolytica (Titanium 5 + PH-M, Elanco Animal Health, Greenfield, IN). Calves were dewormed (Valbezene, Zoetis, Parsippany, NJ) and treated with Cydectin (Bayer, Shawnee Mission, KS) for control of ectoparasites. A completely randomized design was used to stratify calves by dam age, birth date, birth weight, and sex to 1 of 3 backgrounding systems for 55 d after weaning in three different, and separate locations: (1) fed a high roughage ration delivered in a dry lot (DL); (2) grazing perennial pasture vegetation (PP), and 3) grazing summer grown cover crop (CC). All experimental animals received a free-choice mineral (Wind and Rain, Purina Animal Nutrition LLC, MN) throughout the 55-d backgrounding period (Table 1).

At the end of backgrounding, all cattle were placed into a feedlot and delivered a similar finishing high energy ration until harvest. Between backgrounding to finishing, body weight (**BW**) data were collected and average daily gain calculated. Calves were weighed using a hydraulic squeeze chute (Tru-Test XR 3000, Mineral Wells, TX) with load cells mounted under the chute. Cattle had ad libitum access to water and free-choice minerals (Wind and Rain, Purina Animal Nutrition LLC, MN) throughout the 55-d backgrounding period and during finishing. Average daily gain (ADG) was then calculated from periodic BW measurements during backgrounding and finishing phases.

### Backgrounding management and dietary treatments

Dry lot backgrounded calves were fed a high roughage haylage-based ration delivered every morning (0800 h) using a truck-mounted (F-Series, Ford Motor Company, Dearborn, MI) total-mixed ration mixer (KUHN KNIGHT Model Auggie 3136, Brodhead, WI) fitted with a scale with 4.5-kg resolution (Weigh-Tronix, Fairmont, MN). Perennial Pasture (PP) paddocks used in this experiment have historically been managed as rotational pastures. Forage species within PP consisted of a mix of perennial ryegrass (Lolium perenne), quackgrass [*Elymus repens* (L) Gould], orchardgrass [*Dactylis glomerata* (L.)], smooth bromegrass [*Bromus inermis* (L.)], red clover [*Trifolium pretense* (L.)], and alfalfa [*Medicago sativa* (L.)] in various proportions. Each pasture paddock used in the experiment was approximately 2.43 ha. Each pasture paddock was sequentially sampled for total available forage prior to grazing. Stocking rates were set by calculating an estimated forage allowance at 70% utilization. Calves grazing PP were then rotated between multiple pastures to ensure adequate estimated forage allowance. The summer annual CC were seeded using a Great Plains no-till drill (Great Plains, Salina, KS) 60 d (July, 21, 2018) before commencement of the study. Annual CC vegetation consisted of 82% cereal oats (Avena sativa var. Mustang), 7.6% purple top turnips (Brassica rapa subspp. Rapa var. Purple Top), 7.6% Hunter forage brassica (Brassica spp. Var. Brassica rapa subspp. Pekinensis x Brassica rapa subspp. Rapa), and 2.6% Graza forage radish (Raphanus raphanistrum subspp. Sativus var. Graza). Prior to grazing CC calves, the square-shaped study area was divided in half using high-tensile electric fence to allow for strip grazing of the study area. Each strip was established at a pre-set size of 10% of the total study area and was sequentially sampled weekly for total available forage. Stocking rates were then set by calculating an estimated forage allowance at 70% utilization. Calves grazing CC were permitted access to forward strips according to the estimated forage allowance.

### Rumen sample collection

Samples were collected by using an esophageal tubing (Rumen-Mate®, B & B Manufacturing, Sumas, WA). During the entire study period, rumen ingesta samples were collected at weaning (d 0; T1), early backgrounding (d 28; T2), late backgrounding (d 55; T3), early finishing (d 121; T4) and late finishing (d 185; T5). After collection, rumen samples were stored in carefully labeled 50 mL plastic tubes, placed temporarily in a liquid nitrogen tank and transported to the laboratory for storage at − 80° C until DNA extraction.

### DNA extraction and bioinformatics processing

On the day of DNA extraction, individual rumen fluid samples were thawed on ice, then homogenized on a blender (Oster® classic) for 2 min to thoroughly mix liquid and solid fractions. The blend was centrifuged at 10,000*g* × 20 min (Thermo Sorvall ST16R Refrigerated). Supernatant was discarded, the resulting pellet was mixed (vortexed) and then dissolved in four parts of extraction buffer (100 mM Tris/HCl, 10 mM ethylenediaminetetraacetic acid [EDTA], 0.15 M NaCl pH 8.0). Eluates were incubated at 4 °C for one hour to maximize release of particle associated rumen microbes and then centrifuged again at 500*g* for 15 min to discard plant particles. Supernatant was transferred to a new tube and then centrifuged one more time at 10,000*g* for 25 min (4 °C). Supernatant was discarded and the final pellet was used for DNA extraction. DNA was extracted by repeated bead-beating followed by precipitation, elution and purification using columns from the QIAamp® DNA PowerSoil Kit, (Germantown, MD) and following manufacturer’s instructions. After DNA integrity was measured, high quality DNA of 189 samples were used for rumen bacteria community profiling through 16S rRNA amplicon sequencing, targeting the V4 hyper variable region (barcode primer pair 515f-GTGCCAGCMGCCGCGGTAA and 806r-GGACTACHVGGGTWTCTAAT) on the Illumina MiSeq sequencing platform at the University of Minnesota Genomic Center (UMGC). Raw reads were trimmed to remove primers using cutadapt, and filtered to remove low quality reads (less than Q = 30) using fastx_toolkit. High quality reads were considered for downstream analysis using the DADA2 plugin within qiime2 [62], which performs denoising, merging of paired-end reads and removal of chimeric sequences to produce unique amplicon sequence variants (ASVs). Taxonomic assignment of these ASVs was carried out using the trained naïve Bayes classifier on reference sequences (clustered at 99% sequence identity) from Greengenes 13_8 plugins within QIIME2 [63, 64].

### Statistical analyses

Average daily gain data was analyzed with the use of SAS 9.4 (SAS Inst., Inc., Cary, NC) as a completely randomized design with individual animals used as experimental units. Data were checked for normality using PROC UNIVARIATE procedure of SAS (SAS Inst., Inc., Cary, NC). Average daily gain data calculated from body weight measurements collected repeatedly throughout the study and were analyzed as repeated measures using the PROC MIXED procedure of SAS (SAS Inst., Inc., Cary, NC). The model included the fixed effects of backgrounding treatment, sampling d and treatment x sampling d interaction while individual animals were considered a random effect. The *P* values were adjusted for multiplicity based on the Tukey–Kramer method. All microbial community ecology analyses were performed within the R statistical interface [65]. Alpha diversity (Shannon, Observed and Simpson) and beta diversity (Bray Curtis distances) were calculated using the R vegan package [66]. Weighted and unweighted UniFRac distances were calculated using the phyloseq package [67]. Permutational multivariate analyses of variance **(**PERMANOVA) test was used to check the significant differences. The false discovery rate (FDR)-adjusted Kruskal–Wallis multiple comparisons (q < 0.05) and species indicator analysis (indicator values, > 0.5; *P* < 0.05) as implemented in the labdsv R package [68], were used to detect taxa differentially abundant at weaning (T1), backgrounding (T2&T3) and finishing (T4&T5). All graphs were made using the stats, vegan and ggplots R packages [69]. Associations between average daily and representative genus was measured using compositionally corrected spearman correlation coefficients within the R psych package [70]. Network visualization and calculation of network attributes were carried out using Cytoscape.

## Supplementary Information

**Additional file 1.** Compositional, taxonomic and microbiome network traits distinguishing calves under each backgrounding system.

**Additional file 2.** Indicator ASVs characterizing calves under each backgrounding system at day 55 of backgrounding.

## Data Availability

Raw 16S rRNA sequence data are available from the Nucleotide Archive under study accession number PRJNA763290.

## Abbreviations

16S rRNA: 16 Svedberg ribosomal ribonucleic acid
ADG: Average daily gain
ANOVA: Analysis of variance
BLAST: Basic local alignment search tool
bp: Base pair
CC: Cover crop
DMI: Dry matter intake
DL: Drylot
DNA: Deoxyribonucleic acid
d: Day
ASV: Amplicon Sequence Variant
PP: Perennial pasture
T1: Weaning
T2: Early backgrounding
T3: Late backgrounding
T4: Early finishing
T5: Late finishing
