# Supplementary material for: Microbiome network traits in the rumen predict average daily gain in beef cattle under different backgrounding systems
Source: Anim Microbiome. 2022 Mar 28;4:25. doi: 10.1186/s42523-022-00175-y (PMC8961956; doi:10.1186/s42523-022-00175-y)
Supplement: Supplementary file 1 — Additional file 1. Compositional, taxonomic and microbiome network traits distinguishing calves under each backgrounding system. [file 42523_2022_175_MOESM1_ESM.pdf]

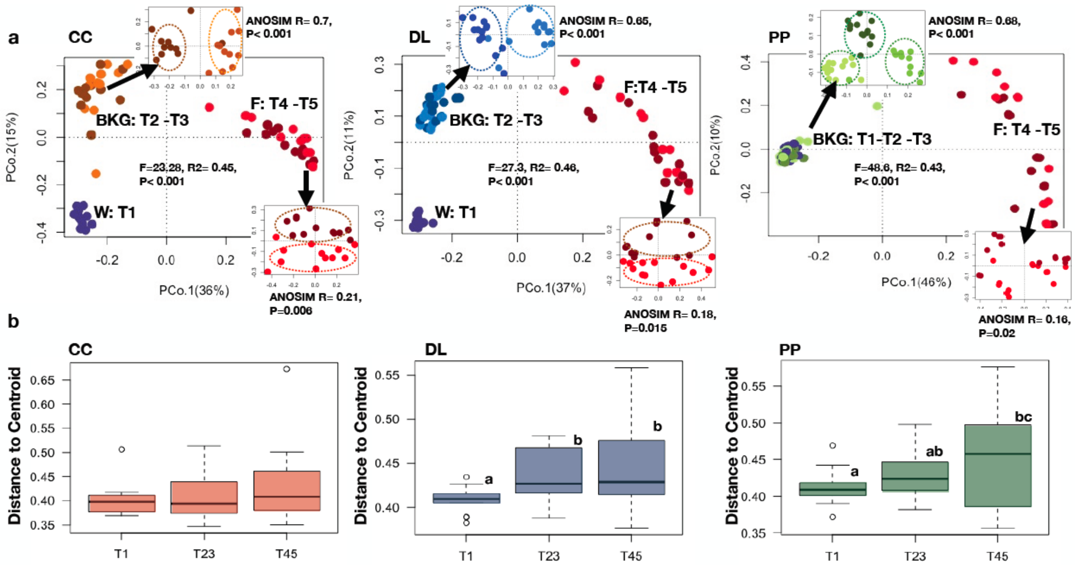

Figure S1. Rumen bacterial composition withing BKG system at weaning (T1), backgrounding (T2 -T3), and finishing (T4 -T5). (a) Principal coordinate analysis (PCoA, Bray-Curtis distances) showing bacterial compositional differences across time points for each, BKG system and finishing. PERMANOVA test results, along with their statistics are displayed next to each PCoA plot. (**b**) Interindividual variation in rumen microbiome composition within each BKG system across time points. Different letters represent significant differences based on Wilcoxon Rank Sum tests (P<0.05). Dry lot (DL); cover crop (CC) and perennial pasture (PP)

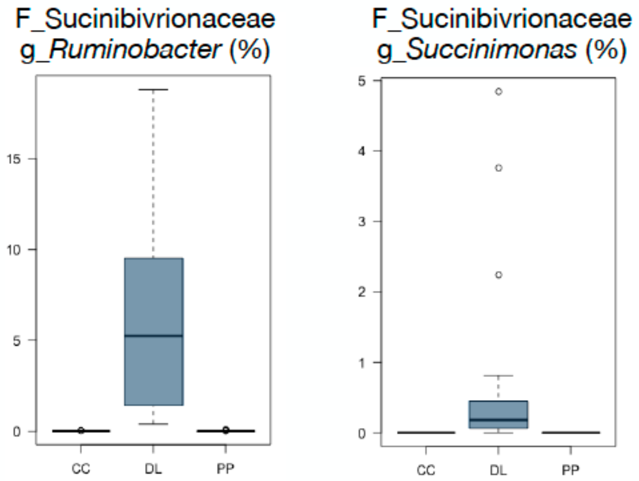

Figure S2. Relative abundance of taxa affiliated to the Succinivibrionaceae across at T2 and T3 of BKG. DL calves show significant higher abundance of *Ruminobacter* and *Succinimonas* compared to CC and PP calves (P<0.001). Dry lot (DL); cover crop (CC) and perennial pasture (PP).

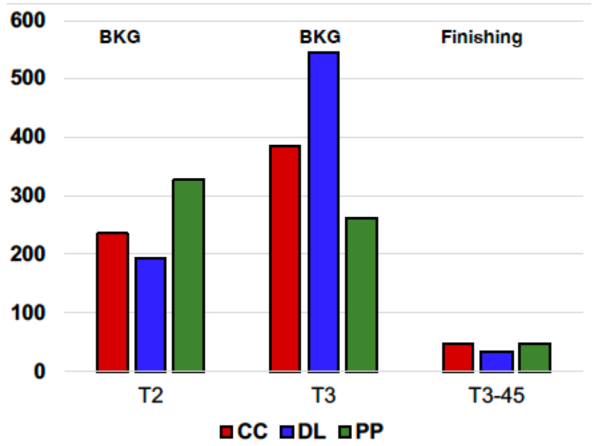

Figure S3. Number of interactions denoting co-abundance between bacterial taxa across time points for each BKG system. All correlations considered are Spearman's correlation coefficient > 0.7, q<0.05. Dry lot (DL); cover crop (CC) and perennial pasture (PP).

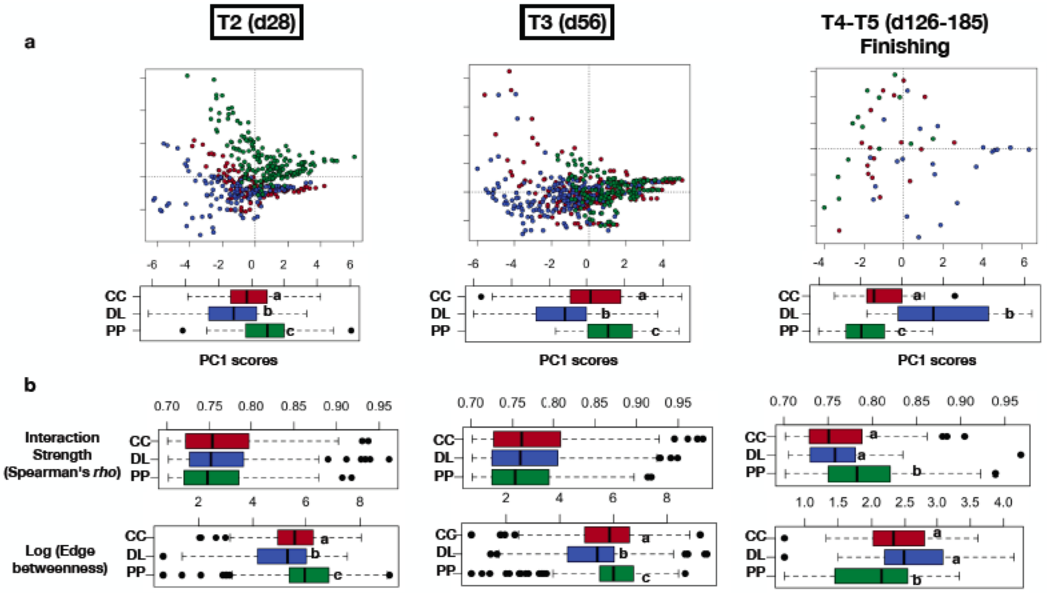

Figure S4. Comparison of overall network topology structure and interactions between BKG systems across timepoints. (a) Principal Component Analyses (PCA) showing differences in network topology considering all network attributes evaluated: Neighbor connectivity, degree, average shortest path length. In the PCA, each symbol corresponds to one amplicon sequence variant (ASV). Boxplots below each PCA show ordination score distribution along PC1. (**b**) Comparison of interaction strength (average Spearman correlation coefficients) and edge betweenness stats. Different letters represent significant differences based on Wilcoxon Rank Sum tests (P<0.05). Dry lot (DL); cover crop (CC) and perennial pasture (PP)
